# Re-Reading

**Published:** 1855-02

**Authors:** 


Re-Reading.—A writer in the Home Journal in reference to the article on *Health, Wealth, and Religion*, in the December No., which has attracted so much attention, does not read aright,—when he construes it to say that "the lovely Moravian Brethren were falling into the habit of selling or renting pews in church." We never knew such a case, the assertion was intended to apply to the Methodists, and to them only.

President ——, of the University of ——, writes us Feb. 1st, that the article above alluded to, ought to be republished in tract form for general distribution. As good as the December number may seem to be, we think that this February number is the best yet issued, and consequently make a "tract" of it ourselves, furnished at large discount to the trade.

